# Acute Promyelocytic Leukemia and Brugada Syndrome: A Report on the Safety of Arsenic Trioxide/All-Trans-Retinoic Acid Therapy

**DOI:** 10.3390/hematolrep15030045

**Published:** 2023-07-17

**Authors:** Giorgio Rosati, Sofia Camerlo, Matteo Dalmazzo, Melissa Padrini, Tiziano Tommaso Busana, Marco De Gobbi, Alessandro Fornari, Alessandro Morotti

**Affiliations:** 1 Department of Clinical and Biological Sciences, San Luigi Gonzaga Hospital, University of Turin, Orbassano, 10043 Turin, Italy; sofia.camerlo@unito.it (S.C.); matteo.dalmazzo@unito.it (M.D.); melissa.padrini@unito.it (M.P.); tizianotommaso.busana@unito.it (T.T.B.); marco.degobbi@unito.it (M.D.G.); alessandro.morotti@unito.it (A.M.); 2 Department of Oncology, Division of Pathology, San Luigi Gonzaga Hospital, Orbassano, 10043 Turin, Italy; a.fornari@sanluigi.piemonte.it

**Keywords:** Brugada syndrome, acute promyelocytic leukemia, arsenic, all-trans-retinoic acid, sudden death

## Abstract

Acute promyelocytic leukemia (APL) is a rare and aggressive form of acute myeloid leukemia (AML). Instead of cytotoxic chemotherapy, a combination of all-trans-retinoic acid (ATRA) and arsenic trioxide (ATO) represents front-line therapy in low-risk patients. However, the therapeutic approach could be challenging in the case of a concomitant diagnosis of Brugada syndrome (BrS), a genetic disease characterized by an increased risk of arrhythmias and sudden cardiac death. Here, we present the case of a BrS patient who has been diagnosed with low-risk APL and treated with ATRA and ATO without observing arrhythmic events. In particular, we highlight the difficulties encountered by clinicians during the diagnostic work-up and the choice of the best treatment for these patients.

## 1. Introduction

Brugada syndrome (BrS) is an inherited disease first described in 1992 and associated with an increased risk of fatal arrhythmic events and sudden death. It has autosomal dominant transmission with incomplete penetrance and is caused by mutations in genes coding for membrane channel proteins. Although more than a hundred mutations are known to be implicated, loss-of-function mutations in the sodium channel SCN5A gene are the most frequent (20% of cases). Currently, the diagnosis of BrS can be established either in the presence of a spontaneous type 1 ECG pattern or in the case of survival from a cardiac arrest—caused by ventricular fibrillation or polymorphic ventricular tachycardia—combined with an induced type 1 ECG pattern. Patients with this disease must avoid several drugs that favor the occurrence of arrhythmic (and possibly fatal) events [1,2,3].

The prevalence of asymptomatic Brugada ECG patterns is up to 9 times more frequent in men than women and is estimated to be 0.05% worldwide, with a maximum of 0.37% in Southeast Asia and a minimum of 0% in North Africa. Symptomatic Brugada syndrome prevalence has been less studied, but a 10% event rate at 2.5 years was demonstrated among patients with a typical ECG pattern [2,4,5].

Acute promyelocytic leukemia (APL) is an aggressive form of acute myeloid leukemia (AML), characterized by a block in the differentiation of myeloid cells at the stage of promyelocytes. According to the World Health Organization (WHO), APL is listed as an AML with the specific acquisition of the translocation t(15;17). Due to this chromosomal abnormality, the retinoic acid receptor alpha (RARA) gene and the promyelocytic leukemia (PML) gene merge to form the PML-RARA fusion protein, which downregulates the transcription of several genes involved in myeloid differentiation and alters self-renewal pathways [6,7,8,9].

It is only in the last decade that the combination of all-trans-retinoic acid (ATRA) and arsenic trioxide (ATO) has become the frontline therapy for APL in low-risk patients in place of cytotoxic chemotherapy. Side effects of arsenic include increased liver enzymes, neurotoxicity with peripheral neuropathy, and prolongation of the QTc interval, which can lead to arrhythmic events [10,11,12].

The European incidence of APL is 0.12/100,000/year, with considerable variability among countries and a peak of 0.26/100,000/year in Spain. APL incidence does not seem to differ between males and females and is highest between the ages of 20 and 60 [13,14,15].

The low frequency of these conditions reflects the paucity of reports we found in the medical literature. In 2013, Sgherza et al. described the case of a 69-year-old man affected by chronic myeloid leukemia who had a syncope event and was diagnosed with BrS after one year of treatment with dasatinib [16]. Matsubara et al. and Avash Das et al. reported in 2004 and 2017, respectively, the appearance of a Brugada pattern in patients with AML and febrile neutropenia [17,18]. In addition to the three works cited above, we did not find any articles describing BrS and APL combined.

Little (if any) is known about the safety of APL treatment in patients affected by BrS. Therefore, we aim to point out the diagnostic and therapeutic challenges the clinician might deal with when these two rare conditions are combined. Here we present the case of a young BrS patient diagnosed with APL who manifested no adverse events during the first cycle of therapy.

## 2. Materials and Methods

This report was conducted according to ethical principles consistent with the Declaration of Helsinki. Since this article used only de-identified patient records and did not involve the collection, use, or transmittal of individually identifiable data, it was exempt from Institutional Review Board approval.

Written informed consent for publication was obtained from the patient.

For the diagnostic and therapeutic work-up, the European Society for Medical Oncology (ESMO) and the National Comprehensive Cancer Network (NCCN) clinical practice guidelines for acute myeloid leukemia (updated as of November 2022) were followed.

The histological diagnosis was conducted according to the World Health Organization’s (WHO) Classification of Myeloid Neoplasms and Acute Leukemia.

The medical literature was investigated by searching both the MEDLINE and EMBASE databases.

To confirm the molecular diagnosis of APL, we extracted nucleic acids with the Maxwell^®^ CSC RNA Blood Kit using the Maxwell^®^ Promega extraction kit, and PCR was performed using the EasyPGX-ready PML-RARA fusion kit from Diatech Pharmacogenetics.

Flow cytometry analyses were carried out with Becton Dickinson FACSLyric™ (12 colors), using lyophilized antibodies from EuroFlow™ Consortium.

For immunohistochemistry, these antibodies were used: polyclonal rabbit anti-human MPO (Dako), polyclonal rabbit anti-human CD117/c-KIT (Dako), and monoclonal mouse anti-human CD34 class-II, clone QBEnd/10, ready-to-use (Dako Omnis). All the analyses were performed on the Dako Omnis platform.

The machine used to perform the ECGs was the Cardiovit AT-102 G2 by Schiller.

## 3. Case Presentation

In November 2022, a 38-year-old Caucasian male was admitted to the emergency department for the appearance of bilateral inguinal swelling. Smoking habits (20 packs per year), former drug addiction (cocaine assumption up to 7 months earlier), and Brugada syndrome were reported in his past medical history. He had been vaccinated against SARS-CoV-2 with three doses of Comirnaty, the last in February 2022, and never suffered from Coronavirus disease.

In previous years, the patient had been tested for BrS due to his family history: the ECG presented a drug-induced type-1 pattern, whereas the 24-h ECG monitoring and the ergometric test were negative. He never had syncopal or arrhythmic events.

At admission, vital signs were found normal, and the physical examination showed only a few movable and painful inguinal lymph nodes associated with signs of genital inflammation. The liver and spleen were not palpable, and no B-symptoms were recorded. No signs of bleeding, diathesis, or thrombosis were present.

Blood counts reported neutropenia (0.47 × 10^3^/μL), lymphopenia (0.36 × 10^3^/μL), low platelet count (86 × 10^3^/μL, confirmed on citrate), and mild anemia (Hb 12.4 mg/dL, MCV 93 fl). Normal serum levels of iron and folic acid and increased vitamin B12 and ferritin levels were recorded. Thus, we ruled out deficiency-caused pancytopenia. Moreover, no signs of hemolysis were present. Other blood tests showed normal kidney function (serum creatinine of 0.9 mg/dL and estimated GFR of 94 mL/min) and electrolyte levels (Na 138.58 mmol/L, K 4.45 mmol/L, Ca 2.37 mmol/L), increased C-reactive protein (6.34 mg/dL), and normal LDH (214 U/L). Liver function enzymes resulted as follows: AST 28 U/L, ALT 64 U/L, GGT 41 U/L, ALP 61 U/L, and total bilirubin 0.7 mg/dL. Disseminated intravascular coagulation was excluded in view of normal coagulation tests: PT ratio 1.11, aPTT ratio 0.85, and fibrinogen 489 mg/dL. The patient tested negative for HIV, HCV, EBV, and CMV antibodies; in addition, serological tests for HBV, including HBsAg, HBcAg, and antibodies, were negative.

**Figure 1:**
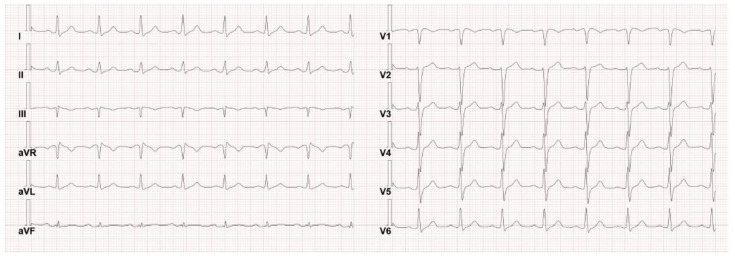
The patient’s basal ECG on admission was found normal, as can happen in inducible type–1 BrS.

Upon admission, a chest X-ray revealed no evidence of pneumonia or other lesions, and the basal ECG was normal (see Figure 1), as can typically happen in inducible type-1 BrS. For this reason, the patient was monitored with telemetry during the first days of hospitalization.

As to what concerns the genital area infection, microbiological tests (urine culture, blood cultures, and T. pallidum antibody assay) resulted in negative results, but the patient responded to the empirical antibiotic treatment with doxycycline 100 mg/day.

Suspecting a hematological disease, a bone marrow biopsy was planned. However, given the patient’s medical history of Brugada type 1 syndrome, we addressed the consultant cardiologist, who suggested avoiding drugs with a proarrhythmic effect (e.g., lidocaine) and performing this invasive procedure under continuous ECG monitoring. The overall arrhythmic risk was assessed to be low, considering the absence of a family history of juvenile sudden death, the absence of malignant syncopes/arrhythmias, and the drug-induced type 1 ECG pattern.

The biopsy was conducted with an easily accessible defibrillator while monitoring the ECG. Intravascular midazolam (6 mg) for sedation and subcutaneous mepivacaine (200 mg) as a local anesthetic were administered. No heart rhythm alterations or other adversities occurred during or after the procedure.

The cytofluorimetric analysis of bone marrow aspirate showed big granular myeloid cells CD11b−, CD11c−, CD13+, CD33+, CD56−, CD117+, HLA-DR−, TdT−, and MPO+ compatible with promyelocytes, amounting to 85% of cells. The flow cytometric results were therefore suggestive of APL.

**Figure 2:**
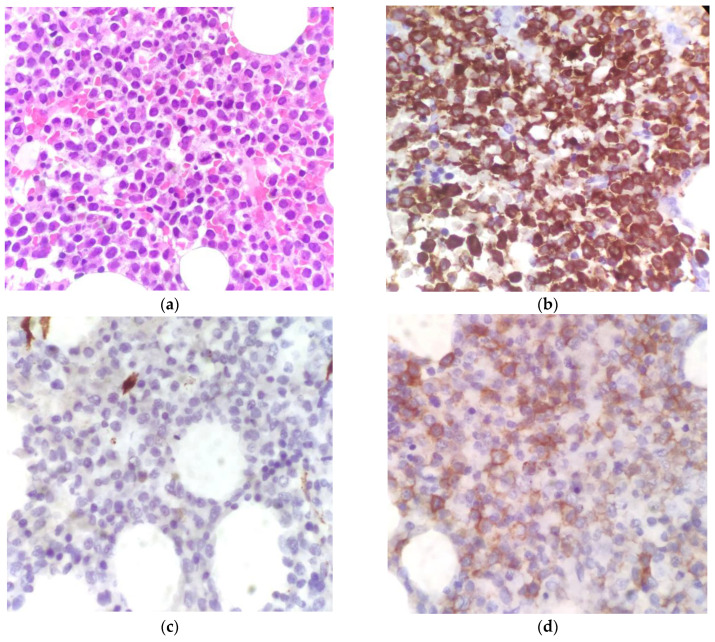
Bone marrow histology. (**a**) H&E stain, ×400 magnification; (**b**) IHC showing myeloperoxidase (MPO) positivity; (**c**) IHC showing CD34 negativity; (**d**) IHC showing weak positivity for CD117.

The histological examination confirmed the suspicion. The bone marrow had 80% to 90% cellularity and was characterized by an immature-looking myeloid population with oval nuclei and hypergranular eosinophilic cytoplasm. At the immunohistochemical assay, the cells showed intense and widespread positivity for MPO and weaker positivity for CD117, but they were negative for CD34, CD56, and TdT (see Figure 2).

Cytogenetic and molecular tests were conducted on bone marrow cells too. The fluorescence in-situ hybridization (FISH) found the translocation t(15;17)(q32;q21), and the reverse transcriptase-polymerase chain reaction (RT-PCR) was positive for the PML-RARA fusion gene (see Table 1).

In light of the newly diagnosed APL, we think that the lack of coagulopathy was likely due to the early identification of the disease and the immediate start of induction therapy.

Given the low white blood cell count, the patient was considered at low risk and therefore was started on ATO 0.15 mg/kg/day intravenously (15.3 mg/day in total) and ATRA 45 mg/m^2^/day per os (50 mg/day in total) divided into two doses for 28 days. The supportive therapy regimen consisted of acyclovir 400 mg twice a day, fluconazole 100 mg/day, and allopurinol 300 mg/day. During the treatment, daily ECG and electrolyte tests, including magnesium, were performed in order to rapidly identify possible alterations in heart rhythm.

Despite the prophylaxis with dexamethasone against the differentiation syndrome, the white blood cell count increased up to 20 × 10^3^/μL in the absence of symptoms, and cytoreductive therapy with hydroxyurea had to be added for ten days. In addition, therapy had to be withheld for two days due to liver toxicity to fluconazole (ALT 512 U/L, AST 217 U/L, LDH 1365 U/L), which was no longer administered. Bone marrow toxicity was mild, and the patient needed to transfuse two units of RBC concentrate just at the end of the treatment. Overall, the therapy was well tolerated, and it was not necessary to withhold it due to QTc prolongation. No arrhythmic or syncopal events occurred.

After the conclusion of the first cycle of therapy, the PML-RARA fusion gene was still identified by RT-PCR on bone marrow cells. However, cytofluorimetric analysis of bone marrow aspirate showed a reduced promyelocyte count to less than 0.1% of myeloid cells. As a result, these findings were consistent with a complete response.

After hematological recovery, the patient was discharged and started outpatient consolidation therapy with ATRA and ATO.

## 4. Discussion

To our knowledge, the safety of APL treatment in Brugada syndrome has never been investigated. Consequently, when the two conditions come together, the assessment of the arrhythmic risk for medications used in the diagnostic and treatment phases remains challenging.

General principles for BrS management as recommended by current guidelines include avoidance of cocaine, cannabis, and excessive alcohol intake, prompt treatment of fever and avoidance of drugs that may induce ST-segment elevation in right precordial leads. With specific regard to the last point, the European Society of Cardiology (ESC) guidelines suggest the brugadadrugs.org site (accessed on 12 January 2023) as a source of consultation [1,3].

Although there is conflicting evidence and divergence of opinion about the safety of lidocaine in BrS, when used for local anesthesia, it seems to be safe if combined with adrenaline and administered in low quantity [10,11,12]. Thus, when evaluating the biopsy, we chose to replace lidocaine with mepivacaine as the local anesthetic since there appear to be no reports about a possible triggering effect. Furthermore, for sedation, we decided to use the benzodiazepine midazolam, considering its short half-life and the good results obtained in other experiences [19,20,21].

Once the diagnosis was established, the safety of supportive treatment and chemotherapy had to be assessed.

In AML patients, posaconazole is the preferred drug for fungal prophylaxis because of its higher efficacy compared to other agents. Nevertheless, fluconazole was used instead in relation to its lower risk of both cardiac events and interaction with ATRA [22,23].

Based on the white blood cell count <10,000 WBC/μL and the absence of symptoms, the patient was considered to be “low-risk”, for which ATRA + ATO is the treatment of choice.

Although QTc prolongation is a common event during treatment with arsenic and exposes the patient to the risk of developing ventricular arrhythmia (i.e., torsade de pointes), various studies showed that significant arrhythmias are rare and can be prevented with ECG monitoring and management of electrolyte disturbances and concomitant medications [24,25]. From 2002 to 2019, Gill et al. monitored for 8 years (range, 8–215 months) a cohort of 129 patients affected by APL in complete remission and in maintenance therapy with ATRA, ATO (10 mg/day PO), and ascorbic acid. Significant QTc prolongation was not observed, and arrhythmic events were not reported [26]. Similarly, from 2001 to 2005, Aribi et al. monitored for more than 36 months (range, 4–55 months) 8 patients with relapsed APL who received induction therapy with ATO (0.15 mg/kg/day IV), consolidation with a combination of ATO (0.15 mg/kg/day for a month and repeated every 2 months for 5 courses), ATRA and gemtuzumab-ozogamicin, and maintenance with idarubicin, ATRA, 6-mercaptopurine, and oral methotrexate. In this work, too, cardiac events were not observed by the authors [27].

As far as we know, the safety of arsenic in Brugada syndrome was never studied. Therefore, the alternative induction regimen consisting of anthracyclines was evaluated and excluded in consideration of their action on the ST-T segment, their overall cardiotoxicity, and their systemic effects.

Despite the absence of previous cardiac events in relation to the history of drug addiction, the patient was offered to begin the treatment in a monitored setting, but he refused. Accordingly, he was treated for 28 days in our division and then discharged to continue the outpatient consolidation therapy.

Concerning the consolidation treatment, the alternative regimen to the use of ATO is represented by the combination of ATRA and anthracycline-based chemotherapy. However, this option requires two years of maintenance therapy with methotrexate and 6-mercaptopurine. In our case, ATO was well tolerated, and no arrhythmic events were recorded. Therefore, the consolidation cycle with ATRA and ATO was preferred to the use of anthracyclines [11,12].

As of February 2023, neither arrhythmic events nor ECG alterations were recorded.

## 5. Conclusions

APL and BrS are two rare diseases, and in the literature, no data are available about the safety of APL treatment when both conditions are present. The alternative treatment option to ATO is standard chemotherapy with an anthracycline drug (idarubicin) used in high-risk APL. No arrhythmic risk is associated with idarubicin, but the anthracyclines could be cardiotoxic and have systemic side effects.

In consideration of the risks and benefits, we have chosen a chemo-free treatment with ATRA and ATO, and no arrhythmic events have been recorded. Our experience, based on a single case, has shown no adverse heart events during the induction treatment with ATRA and ATO in a patient affected by BrS. We do not aim to establish the safety of our therapeutic approach, but we hope this work could be a starting point to fill a gap in the literature and could help clinicians in the difficult management of these two rare diseases.

## Figures and Tables

**Table 1:** The analyses performed on the peripheral blood and the bone marrow at the diagnosis.

**Analyses on Peripheral Blood**	
Complete blood count	WBC 0.87 × 10^3^/μL (neutrophils 0.47 × 10^3^/μL, lymphocytes 0.36 × 10^3^/μL, monocytes 0.04 × 10^3^/μL, eosinophils 0 × 10^3^/μL, basophils 0 × 10^3^/μL), PLTs 86 × 10^3^/μL confirmed on citrate, hemoglobin 12.4 mg/dL, MCV 93 fl, hematocrit 36%.
Blood tests	PT ratio 1.11, aPTT ratio 0.85 and fibrinogen 489 mg/dL, creatinine 0.9 mg/dL (eGFR 94 mL/min), sodium 138.6 mmol/L, potassium 4.45 mmol/L, calcium 2.37 mmol/L, glucose 121 mg/dL, AST 28 U/L, ALT 64 U/L, GGT 41 U/L, ALP 61 U/L, total bilirubin 0.7 mg/dL, LDH 214 U/L, uric acid 5.6 mg/dL, C-reactive protein (6.34 mg/dL), erythrocyte sedimentation rate 93 mm/h, iron 55 μg/dL, transferrin 204 mg/dL, ferritin 522 ng/mL, folic acid 16.6 ng/mL, vitamin B12 > 2000 pg/mL, D-dimer 14,187 ng/mL. Negative serological tests for HBV, HCV, HIV, EBV, and CMV.
**Analyses on Bone Marrow**	
Flow cytometry	Big granular myeloid cells CD11b−, CD11c−, CD13+, CD33+, CD56−, CD117+, HLA-DR−, TdT−, and MPO+ amounting to 85% of cells, compatible with promyelocytes.
Histology	Cellularity of 80% to 90% characterized by an immature-looking myeloid population with oval nuclei and hypergranular eosinophilic cytoplasm. At the immunohistochemical assay, the cells showed intense and widespread positivity for MPO and weaker positivity for CD117, and they were negative for CD34, CD56, and TdT.
Molecular and cytogenetic analyses	Normal male karyotype at the cytogenetical analysis; presence of t(15;17) at the fluorescence in-situ hybridization (FISH); and PML-RARA fusion gene identified at the RT-PCR.

## Data Availability

There is no new data available.
